# Xylanase (GH11) from *Acremonium cellulolyticus*: homologous expression and characterization

**DOI:** 10.1186/s13568-014-0027-x

**Published:** 2014-04-01

**Authors:** Masahiro Watanabe, Hiroyuki Inoue, Benchaporn Inoue, Miho Yoshimi, Tatsuya Fujii, Kazuhiko Ishikawa

**Affiliations:** 1 Biomass Refinery Research Center, National Institute of Advanced Industrial Science and Technology (AIST), 3-11-32 Kagamiyama, Higashi-Hiroshima, Hiroshima 739-0046, Japan

**Keywords:** Acremonium cellulolyticus, Xylanase, Hemicellulose, Homologous expression, Biomass, Xylan

## Abstract

Cellulosic materials constitute most of the biomass on earth, and can be converted into biofuel or bio-based materials if fermentable sugars can be released using cellulose-related enzymes. *Acremonium cellulolyticus* is a mesophilic fungus which produces a high amount of cellulose-related enzymes. In the genome sequence data of *A. cellulolyticus,* ORFs showing homology to GH10 and GH11 xylanases were found. The xylanases of *A. cellulolyticus* play an important role in cellulolytic biomass degradation. Search of a draft genome sequence of *A. cellulolyticus* for xylanase coding regions identified seven ORFs showing homology to GH 11 xylanase genes (*xylA*, *xylB*, *xylC*, *xylD*, *xylE*, *xylF* and *xylG*). These genes were cloned and their enzymes were prepared with a homologous expression system under the control of a glucoamylase promoter. Six of the seven recombinant enzymes were successfully expressed, prepared, and characterized. These enzymes exhibited optimal xylanase activity at pH 4.0 – 4.5. But this time, we found that only XylC had enormously higher relative activity (2947 U•mg ^−1^) than the other xylanases at optimum pH. This result is surprising because XylC does not retain a carbohydrate-binding module 1 (CBM-1) that is necessary to bind tightly own substrate such as xylan. In this study, we discuss the relationship between activity, pH and sequence of seven xylanases in *A. cellulolyticus*.

## Introduction

Lignocellulosic biomass can be converted into biofuel or bio-based materials (Deutschmann and Dekker [2012]; Kumar et al. [2008]) but must be converted into fermentable sugars by saccharification. Xylan is one of the major structural components of plant cell walls and is the second most abundant renewable biomass resource (York and O’Neill [2008]). Xylan consists of a backbone of β-1,4-D-xylan with short side chains of O-acetyl, β-L-arabinofuranosyl, D-α-glucuronic acid and phenolic acid (Coughlan and Hazlewood [1993]). Xylanases (endo-1,4-β-xylanases; EC 3.2.1.8) catalyze the hydrolysis of the β-1,4 bonds of xylan and thus are important enzymes for the degradation of hemicellulosic polysaccharides (Collins et al. [2005]; Prade [1996]). Based on their amino acid sequence similarities, xylanases are mainly classified into families 10 and 11 of the glycoside hydrolases (GH; http://www.cazy.org/Glycoside-Hydrolases.html; (Coutinho and Henrissat [1999]). GH10 xylanases generally have a molecular weight ≥30 kDa and a low p*I*, while GH11 xylanases are generally smaller (approximately 20 kDa) and have a high p*I* (Beaugrand et al. [2004]). The crystal structures of xylanases show that GH10 enzymes fold into a (β/α)_8_-barrel (Dimarogona et al. [2012]; Harris et al. [1994]; Lo Leggio et al. [1999]), whereas family 11 enzymes have a β-jellyroll structure (Paës et al. [2012]; Sidhu et al. [1999]). Cellulose and xylan are closely linked together in plant cell walls (Carpita and Gibeaut [1993]). Thus, cellulases and hemicellulases work coordinately in the enzymatic degradation of these polysaccharides.

Filamentous fungi produce a wide spectrum of degradation enzymes for cellulose and xylan (van den Brink and de Vries [2011]). *Acremonium cellulolyticus*, isolated by Yamanobe et al., is a high cellulolytic enzyme-producing fungus (Yamanobe et al. [1987]). Fujii et al. reported that the culture supernatant from *A. cellulolyticus* has a higher cellulase specific activity and yields more glucose from lignocellulosic materials than the culture supernatant from *Trichoderma reesei* (Fujii et al. [2009]). Thermostable xylanase from *A. cellulolyticus* was found by Mitsuishi et al. (Mitsuishi et al. [1987]), but this enzyme has not been identified. Furthermore, there is no report of the expression and characterization of the xylanases (GH11) from *A. cellulolyticus*. The cellulose-induced xylanases of *A. cellulolyticus* play an important role in the cellulolytic biomass degradation process. In this study, we searched the xylanase coding regions from the draft genome sequence of *A. cellulolyticus* (unpublished data) and attempted to clarify the characteristics of the all xylanases (GH11).

## Materials and Methods

### Materials

Birch-wood xylan was purchased from Sigma-Aldrich (St. Louis, MO, USA). All other chemicals were of the highest grade commercially available.

### Strain and culture conditions for Acremonium cellulolyticus

*Acremonium cellulolyticus* CF-2612 and Y-94 (CBS136886) were maintained on potato dextrose agar plates (Fang et al. [2009]). The *A. cellulolyticus* YP-4 uracil autotroph was maintained on potato dextrose agar plates containing uracil and uridine at final concentrations of 1 g/l each (Inoue et al. [2013]). Transformants of *A. cellulolyticus* YP-4 were maintained on MM agar plates (Fujii et al. [2012]). For measurement of gene expression, the strains were cultivated in 50 ml of basic medium (24 g/l of KH_2_PO_4_, 1 g/l of Tween 80, 5 g/l of (NH_4_)_2_SO_4_, 1.2 g/l of MgSO_4_•7H_2_O, 0.01 g/l of ZnSO_4_•7H_2_O, 0.01 g/l of MnSO_4_•6H_2_O, 0.01 g/l of CuSO_4_•7H_2_O; pH 4.0) supplemented with 20 g/l soluble starch (Wako Pure Chemical Industries, Osaka, Japan) as a carbon source in 500-ml Erlenmeyer flasks at 30°C for 96 h on a rotary shaker operated at 230 rpm. The cells were centrifuged for 15 min at 3,500 rpm, then the supernatants were centrifuged for 10 min at 13,500 rpm. The pooled enzyme solution was filtered through a 0.45 μM membrane and adjusted with acetate buffer (pH 5.0) containing 0.01% NaN_3_. The samples were stored at 4°C until use.

### Cloning of xylanases and construction of the expression vector

A draft genome sequence of *A. cellulolyticus* (unpublished data) was searched for the xylanase (GH11) genes using *In silico* Molecular Cloning gene analysis software (In Silico Biology, Inc., Yokohama, Japan) based on the internal amino acid sequence. Construction of the xylanase expression vector for *A. cellulolyticus* basically followed a procedure described previously (Inoue et al. [2013]). The genomic regions encoding xylanases were amplified by PCR from CF-2612 chromosomal DNA using primers as indicated in Table 1. The seven amplified xylanase genes were constructed by introducing the appropriate fragment digested with *Hpa*I/*Sbf*I (*xylA*, *xylB*, *xylC*, *xylD*, *xylE* and *xylF*) into the *Eco*RV/*Sbf*I (*xylG*) site of a series of pANC expression vectors containing a glucoamylase (*glaA*) promoter and terminator (Inoue et al. [2013]): pANC209 (*xylA*), pANC210 (*xylB*), pANC223 (*xylC*), pANC230 (*xylD*), pANC231 (*xylE*), pANC232 (*xylF*) and pANC233 (*xylG*) (Table 2).

**Table 1:** Summary of nucleotide primers used in this study

**Primer**	**Nucleotide sequence (5’ → 3’)**
*For plasmids construction*	
AC-F12 (*xylA*)	ATTGTTAACAAGATGAAGATCACATCAGTGTTCG
AC-R12 (*xylA*)	AATCCTGCAGGTTAAGATACAGTAACAGTGGCACTTC
AC-F13 (*xylB*)	ATTGTTAACATCATGGGCATCTCATCTATTCTTC
AC-R13 (*xylB*)	AATCCTGCAGGCTATTGGCACTGACTGTAGTAAGCGT
AC-F25 (*xylC*)	ATTGTTAACAAGATGAAGCTCTCTCTGGCTGCAA
AC-R25 (*xylC*)	AATCCTGCAGGCTAGGACACGGTGATGGTACTAGAAC
AC-F26 (*xylD*)	ATTGTTAACAAGATGCGGTCATTTGCTCGCCTTGTC
AC-R26 (*xylD*)	AATCCTGCAGGTCAGCTAACAGTAAAATCCAGGTAAC
AC-F27 (*xylE*)	ATTGTTAACAAGATGATTTATTTCCCTCAGCTCATG
AC-R27 (*xylE*)	AATCCTGCAGGCTATTGAGTGGCAGTCTGCTGGGCA
AC-F28 (*xylF*)	ATTGTTAACAAGATGTTCTCTTTCAGTACTGCCTT
AC-R28 (*xylF*)	AATCCTGCAGGCTACAAGCATTGATAGTAGTACGGGT
AC-F29 (*xylG*)	ATTGATATCAAGATGGTTGCTTTCTCGAGCTTATTTAC
AC-R29 (*xylG*)	AATCCTGCAGGGTCCAACATCAATGCTACTTACAGC
*For quantitative PCR*	
*xylA*-f	GAGGACGGAGTCAATGGAGA
*xylA*-r	CCGAGAGGTAGGAGCCAGAA
*xylB*-f	TGCTCTCGGTGTTGATGTTG
*xylB*-r	GTGGTCTGGTAGTCGGTGGA
*xylC*-f	GTGTCGACCAACCCTCCATC
*xylC*-r	GTCCAAGTGCCTTCCATGCT
*xylD*-f	TTTAGCGATAGCGGCAGGTT
*xylD*-r	CCCATGTAAGGGAGCGAGTT
*xylE*-f	CGGGCCACAAACTATATCCA
*xylE*-r	TTCCAGCCAACACCAACAAC
*xylF*-f	ACTAGCAAGGACGGCGTAGA
*xylF*-r	CACCGAGGAACTCAGACGAA
*xylG*-f	CAGCACGGGGTTTGAGGTT
*xylG*-r	CCGAAGTTGATGGGAGTGGT
*gpdA*-f	AACATCATTCCCAGCAGCAC
*gpdA*-r	CGGCAGGTCAAGTCAACAAC

**Table 2:** **Summary of seven xylanases (GH11) in** ***A. cellulolyticus***

**Strain**	**Plasmid**	**Xyl11**	**AA**	**MW (Da)**	**CBM-1**	**N-terminal sequence (5 aa)**
Y209	pANC209	A	209	22377	-	AGGIN
Y210	pANC210	B	282	29510	CBM	AEAIN
Y223	pANC223	C	223	23955	-	QSITT
Y230	pANC230	D	190	21029	-	-
Y231	pANC231	E	234	25788	-	ATNYI
Y232	pANC232	F	276	29210	CBM	NTPNS
Y233	pANC233	G	233	24906	-	SAINY

### Homologous expression of the recombinant xylanases

Protoplasts of *A. cellulolyticus* YP-4 were transformed with pANC209, 210, 223, 230, 231, 232 and 233 by nonhomologous integration into the host chromosomal DNA (Fujii et al. [2012]). Gene integration into prototrophic transformants was verified by genomic PCR. The expression of each recombinant xylanase was carried out using the following cultures: Y209 (YP-4 transformed with pANC209; XylA), Y210 (YP-4 transformed with pANC210; XylB), Y223 (YP-4 transformed with pANC223; XylC), Y230 (YP-4 transformed with pANC230; XylD), Y231 (YP-4 transformed with pANC231; XylE), Y232 (YP-4 transformed with pANC232; XylF), Y233 (YP-4 transformed with pANC233; XylG) (Table 2). All cultures were grown in medium containing 20 g/l soluble starch and 5 g/l urea using the method described previously (Inoue et al. [2013]). The recombinant xylanases expressed were purified from the culture supernatant.

### Purification of the recombinant xylanases

*A. cellulolyticus* Y209, 210, 223, 231, 232 and 233 were grown at 30°C on a starch-induced medium. The purification procedures were basically as previously reported (Lee et al. [2009]). The purified enzyme was concentrated, dialyzed against 20 mM sodium acetate buffer (pH 5.0) using a Vivaspin 20 concentrator (10,000 MWCO, Sartorius AG, Goettingen, Germany) and stored at 4°C until use. The purity and size of the protein was analyzed by SDS-PAGE using precast NuPAGE 4 - 12% polyacrylamide Bis-Tris gels (Life Technologies, Carlsbad, CA, USA) (Hachmann and Amshey [2005]). All proteins were identified by N-terminus sequence analysis.

### Protein assay

Protein concentration was determined by a Pierce BCA Protein Assay Kit (Pierce, Rockford, IL, USA) using bovine serum albumin as the standard (Shibuya et al. [1989]; Smith et al. [1985]). Protein solution (25 μl) was mixed with 200 μl of BCA reagent and then incubated at 37°C for 30 min. Protein concentration was determined by measuring the absorption at 570 nm.

### N-terminus sequence analysis

The N-terminal sequences of six xylanases (XylA, XylB, XylC, XylE, XylF and XylG) were commercially determined by Edman degradation using Procise 494 HT (ABI, Foster City, California, USA). Each enzyme was adjusted to 2.0 mg/ml (600 μg) in 20 mM sodium acetate buffer (pH 5.5) prior to degradation.

### Enzyme assay

The xylanase activity assay was based on the method described by Bailey et al. ([1992]), using 1% (w/v) birch-wood xylan (Sigma-Aldrich) as the substrate. Activity was examined under a variety of buffer conditions between pH 3.0 and 8.0 (McIlvaine [1921]). The appropriately diluted protein solution was mixed with 0.45 ml of substrate, followed by incubation for 10 min at 50°C. The reducing sugars produced in the reaction mixture were measured by a 3,5-dinitrosalicylic acid (DNS) assay. One unit of xylanase activity was defined as the quantity of enzyme required to liberate 1 μmol of xylose equivalent per minute at 50°C.

### Differential scanning fluorimetry (DSF)

A series of DSF experiments generally followed the protocol published by Niesen and others (Niesen et al. [2007]; Lo et al. [2004]). The experiments were carried out with a CFX96 Real-Time PCR System^™^ (Bio-Rad) using 450/490 excitation and 560/580 emission filters in a 96-well plate format. Each enzyme was prepared at a final concentration of 0.1 mg/ml (2 μg) in 50 mM sodium acetate pH 4.0 buffer. SYPRO orange dye [5,000-fold stock solution in dimethyl sulfoxide (DMSO); Invitrogen, Carlsbad, CA, USA] was added in twentyfold dilution to the enzyme (Senisterra and Finerty [2009]). The negative control comprised buffer and the SYPRO mixture, without xylanase. Each sample (20 μl per well) was measured between 25 and 90°C using a stepwise gradient of 0.5°C per 5 s. Following curve fitting of the data to the Boltzmann equation, the melting temperature (*Tm*) of each xylanase was calculated using Bio-Rad CFX Manager software (Bio-Rad). The results are shown in Table 3.

**Table 3:** Specific activity and thermal shift assay of six xylanases (GH11)

**Enzyme (GH11)**	**Specific activity at pH 5.5 (U/mg)**	**Specific activity at optimum pH (U/mg)**	**Tm (°C) at pH 5.5**	**Tm (°C) at optimum pH**	**Δ°C**
XylA	320	999 (pH 4.0)	55	59	4
XylB	252	667 (pH 4.0)	62.5	66	3.5
XylC	1434	2947 (pH 4.0)	58	61.5	3.5
XylE	43	304 (pH 4.0)	59	65.5	6.5
XylF	16	34 (pH 4.0)	54	60	6
XylG	35	285 (pH 4.0)	55	64.5	9.5

### Real-time quantitative PCR

Total RNA was extracted from disrupted fungal cells. Single-stranded cDNA was synthesized and then real-time quantitative PCR was conducted as described previously (Fujii et al. [2010]). The expression of each gene was normalized against that of the glyceraldehyde-3-phosphate dehydrogenase gene (*gpdA* accession number AB847425). Results are shown as relative expressions. The gene-specific primers used were shown in Table 1. The nucleotide sequences of each gene from CF-2612 will appear in the GenBank/EMBL/DDBJ nucleotide database under accession nos. AB847990 (*xylA*), AB847991 (*xylB*), AB847992 (*xylC*), AB847993 (*xylD*), AB847994 (*xylE*), AB847995 (*xylF*), and AB847996 (*xylG*).

## Results

### Cloning of xylanase genes from A. cellulolyticus

**Figure 1:**
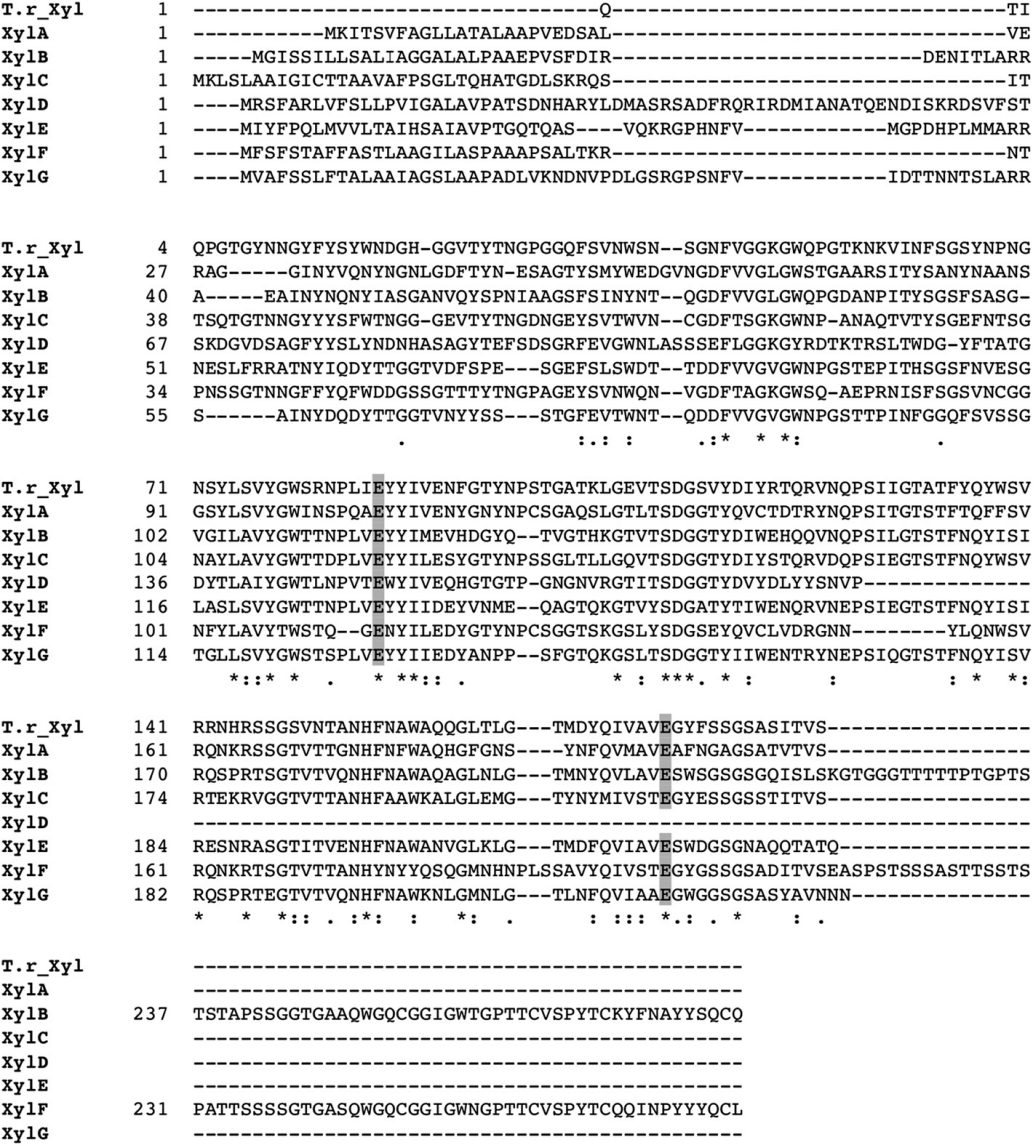
**Sequence alignment between*A. cellulolyticus*GH11s and*T. reesei*GH11.** All seven xylanases (XylA-G) were detected from *A. cellulolyticus*. Strictly conserved residues are highlighted with an asterisk and similarly conserved residues are marked as a colon. The two glutamate catalytic residues are strictly conserved (highlighted with gray background). All xylanases have a signal peptide at the N-terminus whereas only XylB and XylF have a CBM at their C-terminal end. The figure was made using ClustalW Thompson et al. ([1994]).

A search of the genome sequence database of *A. cellulolyticus* identified seven ORFs exhibiting homology to xylanase (GH11) genes: *xylA* (689 bp), *xylB* (915 bp), *xylC* (735 bp), *xylD* (845 bp), *xylE* (760 bp), *xylF* (952 bp) and *xylG* (761 bp). The PCR-amplified DNA fragments were ligated into the expression vectors containing a glucoamylase (*glaA*) promoter and terminator, as described in Materials and Methods. All ligated gene fragments and their ligation sites were verified by sequencing. The *xylD* gene was found to be smaller than the other xylanase genes (Figure 1) and the speculative conserved active site residue (Glu) near the C-terminus of xylanase was absent.

### Homologous expression and purification of the recombinant xylanases

**Figure 2:**
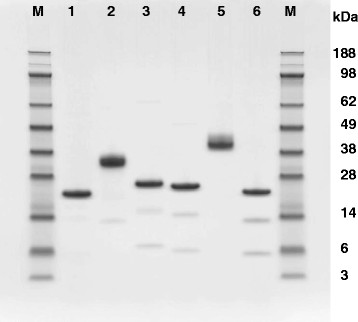
**SDS-PAGE analysis of six kinds of xylanase (GH11).** Lanes: *M*. protein marker, *1*. XylA, *2*. XylB, *3*. XylC, *4*. XylE, *5*. XylF, *6*. XylG. All of these proteins are final products after purification on a Resource ISO column.

For *xylA*, xylB, *xylC*, *xylD*, *xylE*, *xylF* and *xylG* genes, the homologous expression vector (pANC209, 210, 223, 230, 231, 232 and 233, respectively) was transformed into *A. cellulolyticus* YP-4 to provide Y209, Y210, Y223, Y230, Y231, Y232 and Y233 (Table 2). SDS-PAGE analysis showed that the molecular size of XylA (22.3 kDa), XylB (29.5 kDa), XylC (23.9 kDa), XylE (25.7 kDa), XylF (29.2 kDa) and XylG (24.9 kDa) was in accordance with the molecular size calculated from the individual sequences (Figure 2). XylD activity was not detetcted in this expression system because of its incomplete length (Figure 1). Presumably the higher molecular masses of XylB and XylF are largely due to the presence of a CBM-1 with each catalytic domain (Figure 2).

### Comparison of sequence identity and N-terminal sequence analysis

The deduced amino acid sequence of the ORFs indicated that XylA, XylB, XylC, XylD, XylE, XylF and XylG are encoded by 209, 282, 223, 190, 234, 276 and 233 amino acids, respectively, including the individual signal peptides at the N-terminus. Table 2 shows the five N-terminal amino acids of each expressed xylanase. In this homologous expression system, these signal peptides were cleaved off by a signal peptidase on the N-terminal side of the arginine residue. Both XylB and XylF include a carbohydrate-binding module (CBM-1) at their C-terminus. Figure 1 shows a sequence alignment of the active domain in the xylanases. Six xylanases (XylA, XylB, XylC, XylE, XylF and XylG) exhibit amino acid sequence homology of around 11 ~ 52%, with the two catalytic glutamates being strictly conserved.

### Specific activity and the effect of pH on xylanase activity

**Figure 3:**
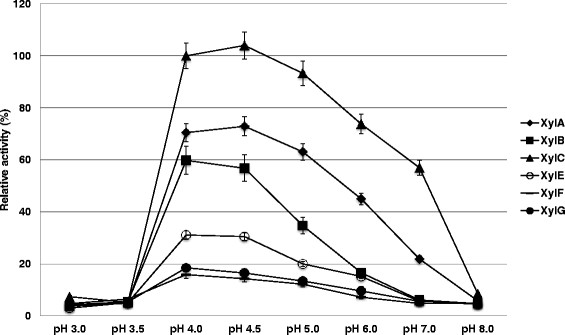
**Optimum pH of six kinds of xylanase (GH11).** The optimal pH of each xylanase was determined using the DNS method with 1% (w/v) birch-wood xylan. The buffers were: 0.1 M citric acid buffer pH 3.0 – 3.5; 0.1 M sodium acetate pH 4.0 − 4.5; 0.1 M sodium citrate pH 5.0 – 6.0; 0.1 M Hepes-NaOH pH 7.0; 0.1 M Tris–HCl pH 8.0. The enzymes are: XylA (black diamond), XylB (black square), XylC (black triangle), XylE (white circle), XylF (black dash) and XylG (black circle). The percent relative activity is based on the activity of XylC at pH 4.0. The data plotted are averages of triplicate experiments.

Xylanase activity assays were carried out using 1% (w/v) birch-wood xylan under a variety of buffer conditions between pH 3.0 and 8.0. Calculation of the specific activity of each xylanase showed that XylC has much higher specific activity, ranging from about 4.5 to 90-fold higher than the other xylanases at pH 5.5 (Table 3). The optimum pH of all the xylanases is between pH 4.0 – 4.5, although XylA and XylC retain activity between pH 4.0 – 6.0 and pH 4.0 – 7.0, respectively (Figure 3). XylC exhibited twofold higher activity at its optimum pH (pH 4.0) than at pH 5.5.

### Thermostability of the xylanases

Fluorescence-based thermal shift assays of the xylanases were performed in a 96-well plate format using a real-time PCR detection system and a volume of 20 μl per well. The samples were heated at 0.1°C/s from 25 to 90°C. All the xylanases were found to be thermostable between 52 and 61°C at pH 5.5, and their thermostability increased by 4 to 10°C at pH 4.0 (Table 3). These results show that all xylanases are in a more active and thermostable form at pH 4.0.

### Gene expression in cultures grown on cellulose and xylan

**Figure 4:**
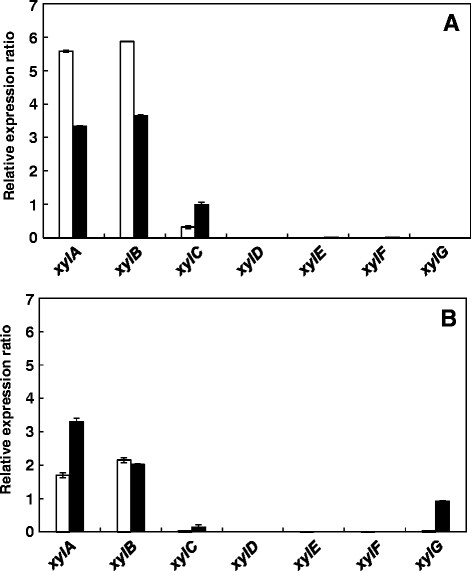
**Expression of xylanase genes.***A. cellulolyticus* Y-94 was cultured in medium containing 50 g/l of cellulose **(A)** or 50 g/l of xylan **(B)**. Culture time was 24 h (data not shown), 72 h (white bars), and 120 h (black bars). Expression levels are shown relative to that of *gpdA* as an internal control.

*A. cellulolyticus* Y-94 was cultured on Solka Floc or xylan as the carbon source for 24, 72, and 120 h, then the levels of expression of their xylanase gene were measured by real-time quantitative PCR (Figure 4). No expression of any xylanase gene was observed after 24 h under both growth conditions (undetectable). A higher expression level of *xylA* and *xylB* was observed in 72 h, indicating that these genes contribute to xylan degradation by *A. cellulolyticus. xylC*, which showed the highest xylanase activity, was expressed slightly compared with *xylA* and *xylB*; the expression of *xylD*, *xylE*, *xylF* and *xylG* was not detected under any of the conditions tested, with the exception of *xylG* at 120 h, xylan cultivation. Figure 4 shows the expression levels relative to that of *gpdA* used as an internal control.

## Discussion

Analysis of the *A. cellulolyticus* genome identified seven ORFs exhibiting homology to the xylanase (GH11) genes: *xylA*, *xylB*, *xylC*, *xylD*, *xylE*, *xylE* and *xylG*, which encode XylA (209 aa), XylB (282 aa), XylC (223 aa), XylD (190 aa), XylE (234 aa), XylF (276 aa) and XylG (233 aa), respectively. Each gene also encodes the signal peptide at the N-terminus. N-terminal sequence analysis showed that each signal peptide is cleaved by an aminopeptidase N-terminal to the arginine residue in this homologous expression system (Inoue et al. [2013]). In addition, Blast alignments indicated that only XylB and XylF have a carbohydrate-binding module (CBM-1) at their C-terminus, suggesting that XylB and XylF bind more strongly to their substrate than the other xylanases. Figure 1 shows the sequence alignment of the active domain in the xylanases. Six xylanases (XylA, XylB, XylC, XylE, XylF and XylG) exhibit amino acid sequence homology of around 11 ~ 52% and strictly conserve the two catalytic glutamate residues, and also strongly conserve other resides located in the speculative catalytic cleft (Paës et al. [2012]). Although the structure of XylC or any of the other xylanases has not been determined, it is expected to have a β-jellyroll structure based on multiple sequence alignment and molecular weight analyses. This protein folding motif is extremely common in small molecular weight (<30 kDa) proteins like the GH11 family (Paës et al. [2012]). Analysis of the *A. cellulolyticus* genome showed the *xylD* gene to be smaller than the other family members (Figure 1), and that it lacks some of the regions conserved in the other xylanases, including one of the catalytic glutamic acids. It seems that the *xylD* gene cannot translate normal xylanase protein (XylD) in *A. cellulolyticus*. Amino acid sequence alignments suggest that the active domains of six xylanases (Xyl11A, B, C, E, F, and G) in *A. cellulolyticus* have common activity and structure, and share high homology with the GH11 family of *Trichoderma reesei,* which is well known for its hyper-productivity (Zou et al. [2012]). There is over 50% sequence identity between XylC and *T. reesei* GH11.

Six of the seven xylanases (Xyl11A, B, C, E, F, and G) were expressed and purified successfully in *A. cellulolyticus* transformant strains. SDS-PAGE analysis shows that the purified XylB, XylE, XylF and XylG are accompanied by one or two smaller, faint bands (Figure 2). However, these proteins exhibited the significant xylanase activity toward the birch-wood xylan (Bailey et al. [1992]). Therefore, their faint bands suggest the nick regions of the recombinant proteins expressed. The nick regions seem to be located somewhere in these folded proteins (unclear). The N-terminus of the expressed protein giving rise to each major band was also determined (Table 2). These data indicate that *A. cellulolyticus* efficiently translates and expresses these xylanase genes using this homologous expression system (Inoue et al. [2013]).

Activity assays conducted at various pH values showed that all the xylanases have their optimum pH between pH 4.0 – 4.5, although XylA and XylC retain activity between pH 4.0 – 6.0 and pH 4.0 – 7.0, respectively (Figure 3). In addition, we examined the specific activity of all the xylanases against xylan and found that XylC has much higher specific activity than the other xylanases (Table 3). XylC exhibited between 4.5 to 90-fold higher activity at pH 5.5 than the other xylanases, and twofold higher activity at its optimum pH (pH 4.0) than at pH 5.5. These results clearly show that XylC plays the important role as a glycoside hydrolase 11 (GH11) of all the xylanases in *A. cellulolyticus*. It is possible that XylC uses a different mechanism for substrate recognition and catalysis compared to other xylanases. Detailed structural studies will be required to clarify this issue.

The levels of expression of the seven xylanase genes in Y-94 cultured for 24, 72, and 120 h were measured by real-time quantitative PCR (Figure 4). No expression of any xylanase gene was observed after 24 h (data not shown). A higher expression level of *xylA* and *xylB* was observed compared to the other xylanases, indicating that these genes contribute to xylan degradation by *A. cellulolyticus* after 72 h. *XylC*, which showed the highest xylanase activity, was expressed slightly less than *xylA* and *xylB*. This result suggests that expression of *xylA* and *xylB* is important for the saccharification of biomass, and that the detailed substrate specificity of *xylA* and *xylB* may be different from that of *xylC*. The expression of *xylD*, *xylE*, *xylF* and *xylG* was not detected under any of the conditions tested, with the exception of *xylG* at 120 h, xylan cultivation (Figure 4).

Thermal shift assays conducted using differential scanning fluorimetry showed that the xylanases have a T_m_ of around 60°C at pH 5.5, and that their thermostability increases by up to 10°C at pH 4.0 (Table 3). These results show that all GH11s in *A. cellulolyticus* are most stable and active at pH 4.0. Industrial use of this enzyme will require thermostability at least up to 70°C. XylC plays an important role in degrading xylan and is a good candidate for industrial use in food, paper and chemical production (Deutschmann and Dekker [2012]; Polizeli et al. [2005]). We are currently attempting to crystallize XylC to determine its atomic-level structure by X-ray crystallography.

## Competing interests

The authors declare that they have no competing interests.
